# Whole-Genome Sequences of Four Clinical Isolates of *Mycobacterium tuberculosis* from Tamil Nadu, South India

**DOI:** 10.1128/genomeA.00186-13

**Published:** 2013-06-20

**Authors:** Sujatha Narayanan, Uday Deshpande

**Affiliations:** National Institute for Research in Tuberculosis, Chetpet, Chennai, Indiaa; Labindia-GPOD Research and Training Division, Thane, Indiab

## Abstract

We report the annotated genome sequences of four clinical isolates of *Mycobacterium tuberculosis* from Tamil Nadu, India.

## GENOME ANNOUNCEMENT

The prevalence of ancestral strains of *Mycobacterium tuberculosis* in India has been reported using various genotyping methods (1–5). The current genotyping methods have low discriminatory power, and whole-genome sequencing of clinical isolates of *M. tuberculosis* has now become the preferred approach for genotyping these clinical isolates. The whole-genome sequences of four clinical isolates of *Mycobacterium tuberculosis*, NIRT202, NIRT203, NIRT204, and NIRT206, from the Tamil Nadu state in south India, are reported here.

The paired-end sequencing was performed on an Illumina HiSeq platform. High-quality reads were mapped to the genome of reference strain *M. tuberculosis* H37Rv (accession no. NC_000962.2) using the CLC bio Genomics Workbench.

The NIRT202 genome was generated by aligning 1.34 million paired reads to the reference genome with mapping coverage between 15 and 53×. The annotation of the genome revealed 3,304 genes or coding sequences (CDS) and 156 synonymous and 204 nonsynonymous single-nucleotide polymorphisms (SNPs) (Table 1).

**TABLE 1:** Genomic features of four clinical isolates of *M. tuberculosis* from Tamil Nadu, India

Parameter	NIRT202	NIRT203	NIRT204	NIRT206
Reference assembly				
Reads mapped	97.21%	96.98%	82.22%	96.83%
Not-mapped reads	2.79%	3.02%	17.78%	3.17%
Total reads	1,342,728	6,071,857	3,139,081	1,342,045
Coverage (×)	15–53	15–130	15–741	16–36
Genes/CDS	3,304	3,795	3,556	3,414
Structural variations				
Deletion	10	07	10	12
Insertion	07	1,248	38	09
Complex	05	18	37	08
Synonymous SNPs	156	615	330	615
Nonsynonymous SNPs	204	829	296	829

Mapping of 6.07 million paired reads to the reference genome resulted in the genomic assembly of strain NIRT203. The mapping coverage ranged from 15 to 130×. Annotation of the reference assembled genome revealed 3,795 genes or CDS and the presence of 615 synonymous and 829 nonsynonymous SNPs (Table 1).

Mapping of 3.13 million paired reads to the reference genome resulted in a reference assembly of strain NIRT204. The mapping coverage ranged between 15 and 74×. Annotation of the reference assembly revealed 3,556 genes or CDS and the presence of 330 synonymous and 296 nonsynonymous SNPs (Table 1).

The reference assembly for NIRT206 was generated by mapping 1.34 million paired reads with coverage ranging from 16 to 36×. Annotation of the reference assembly revealed 3,414 CDS or genes and the presence of 615 synonymous and 829 nonsynonymous SNPs (Table 1).

### Nucleotide sequence accession numbers.

The annotated whole-genome sequences have been deposited in GenBank with the accession no. CP004886 (for NITR202), CP005082 (for NITR203), CP005386 (for NITR204), and CP005387 (for NITR206).
